# Stages of NETosis Development upon Stimulation of Neutrophils with Activators of Different Types

**DOI:** 10.3390/ijms241512355

**Published:** 2023-08-02

**Authors:** Vladimir Inozemtsev, Viktoria Sergunova, Nina Vorobjeva, Elena Kozlova, Ekaterina Sherstyukova, Snezhanna Lyapunova, Aleksandr Chernysh

**Affiliations:** 1 Laboratory of Biophysics of Cell Membranes under Critical State, Federal Research and Clinical Center of Intensive Care Medicine and Rehabilitology, V.A. Negovsky Scientific Research Institute of General Reanimatology, Petrovka Street 25c2, 107031 Moscow, Russia; vika_23s82@mail.ru (V.S.); waterlake@mail.ru (E.K.); kmanchenko@yandex.ru (E.S.); snezhanna.lyapunova@yandex.ru (S.L.); amchernysh@mail.ru (A.C.); 2 Koltsov Institute of Developmental Biology, Russian Academy of Sciences, 26 Vavilov Street, 119334 Moscow, Russia; 3 Department of Immunology, Faculty of Biology, Lomonosov Moscow State University, Lenin Hills 1/12, 119234 Moscow, Russia; nvvorobjeva@mail.ru; 4 Department of Medical and Biological Physics, Sechenov First Moscow State Medical University, 119991 Moscow, Russia; 5 General Pathology Department, Federal Research and Clinical Center of Intensive Care Medicine and Rehabilitology, V.A. Negovsky Scientific Research Institute of General Reanimatology, Petrovka Street 25c2, 107031 Moscow, Russia

**Keywords:** neutrophil, neutrophil extracellular trap, NETosis, chromatin, CLSM, A23187, PMA

## Abstract

Before NETs are released, the neutrophil undergoes structural changes. First, it flattens, accompanied by a change in cell shape and rearrangement of the cytoskeleton. Then, nuclear swelling begins, which ends with the ejection of NETs into the extracellular space. We used widefield and confocal fluorescence microscopy to register morphological and structural changes in neutrophils during activation and NETosis. Different types of activators were used, such as NOX-dependent PMA and calcium ionophore A23187. The measurements were performed in a series of sequential stages. In the first stage (30 s after addition of activators and immediately after stimulation of neutrophils), the response of neutrophils to A23187 and PMA exposure was studied. Subsequently, the characteristics of neutrophils in different phases of activation were examined over a longer period of time (30, 60, 120, 180, and 240 min). The specific features of NETosis development were analyzed separately. During the first 30 s, neutrophils appeared to be heterogeneous in shape and structure of the actin cytoskeleton. Characteristic cell shapes included 30″ type 1 cells, similar in shape to the control, with F-actin concentrated in the center of the cytoplasm, and 30″ type 2 cells, which had flattened (spread) shapes with increased frontal dimensions and F-actin distributed throughout the cell. Later, the development of nuclear swelling, the corresponding changes in neutrophil membranes, and NET release into the extracellular space were evaluated. The conditions determining the initiation of chromatin ejection and two characteristic types of decondensed chromatin ejection were revealed. The results obtained contribute to a better understanding of the biophysical mechanisms of neutrophil activation and NETosis development.

## 1. Introduction

Neutrophils are key components of the innate immune system and have the ability to generate Neutrophil Extracellular Traps (NETs), which play an important role in fighting infection and inflammation [1,2,3]. The antimicrobial effect of NETs is due to the restriction of the pathogen distribution, which plays an important role in innate immunity. NETs play an essential role in the pathogenesis of autoimmune and inflammatory disorders, such as systemic lupus erythematosus, rheumatoid arthritis, small vessel vasculitis, and psoriasis [2,3,4]. NETs are also involved in thrombosis, a variety of pulmonary pathologies, chronic rhinosinusitis, sepsis, and malignancies [5,6,7,8,9]. Inadequate NET formation can lead to adverse effects in patients with chronic obstructive pulmonary disease [10] or blood vessel occlusion [5].

NETs are web-like structures composed of antimicrobial proteins contained in neutrophil granules and the neutrophil’s own DNA [1]. NET release leads to neutrophil death as the membrane is eventually disrupted, the nucleus loses its normal structure, and cell integrity is compromised [11].

To date, two distinct mechanisms of NET formation have been identified: NOX-dependent and NOX-independent formation [12]. The classical activator of NOX-dependent NETosis is PMA (phorbol-12-myristate-13-acetate). After PMA enters the cytosol, protein kinase C activity increases, leading to NOX-dependent NET formation [13]. The generated reactive oxygen species (ROS) subsequently destroy the nucleus envelope [14,15]. NOX-independent NET formation has been described in [16,17], which suggested that calcium ionophores, such as ionomycin, can induce NET formation in an NOX-independent manner [18,19,20].

NET formation is accompanied by numerous rearrangements in the cytoskeleton, membranes, organelles, and nuclear chromatin. The successive stages of NETosis have been described in many papers [14,21,22]. As recently shown [23,24], NETosis consists of three consecutive phases driven by entropic chromatin swelling and culminating in membrane rupture and release of NETs. Despite continuous advances in the understanding of the molecular events that accompany NETosis, little is known about the cellular mechanisms of this process [14], especially in the early phase of activation. According to the literature, actin filaments [23,25,26,27], microtubules (MTs) [23,28], and the vimentin intermediate filament (VIF) are disassembled during NETosis at the beginning of the activation process [29]. The special role of actin filaments and their influence on the process of neutrophil activation was also noted both in vitro [29] and in diseases [30].

Therefore, an in-depth understanding of the cellular events controlling NETosis requires a detailed study of the transformation of neutrophil morphology throughout the process [29].

In our study, a comprehensive analysis of the sequential stages of NETosis stimulated by PMA and the calcium ionophore A23187 was performed using fluorescence widefield and confocal microscopy. An investigation of changes in neutrophil morphology was performed at two time points. The first was 30 s after addition of the activators (immediately after stimulation of neutrophils) and the second was from 30 to 240 min after addition of the activators (development of NETosis).

## 2. Results

### 2.1. Activation of Neutrophils by A23187 and PMA (The Question Is, What Are the Statistical Data on Cell Morphology and the Evolution of Cell Activation?)

**Figure 1:**
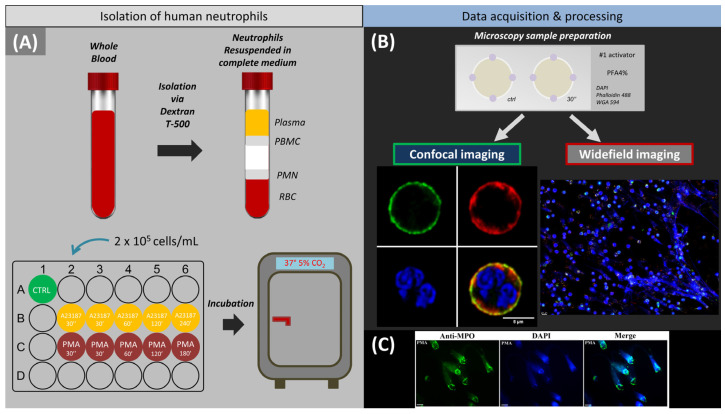
Experimental design. (**A**) Scheme of neutrophil isolation on dextran followed by cell seeding in a 24-well plate and incubation. (**B**) Schematic representation of the sample for fluorescence microscopy and imaging by different fluorescence microscopes followed by software processing. (**C**) Colocalization of decondensed chromatin with myeloperoxidase after neutrophil stimulation with PMA. Blue, staining of chr with DAPI; green, staining of MPO with FITC-conjugated anti-MPO monoclonal antibodies. Scale bar: 25 µm.

A brief scheme of the experiment, including isolation of neutrophils from peripheral blood, activation by A23187 and PMA, and fixation and further analysis of the cells with confocal and widefield microscopy, is presented in Figure 1. NETosis was confirmed by colocalization of chromatin with MPO in NET fibrils (Figure 1C).

**Figure 2:**
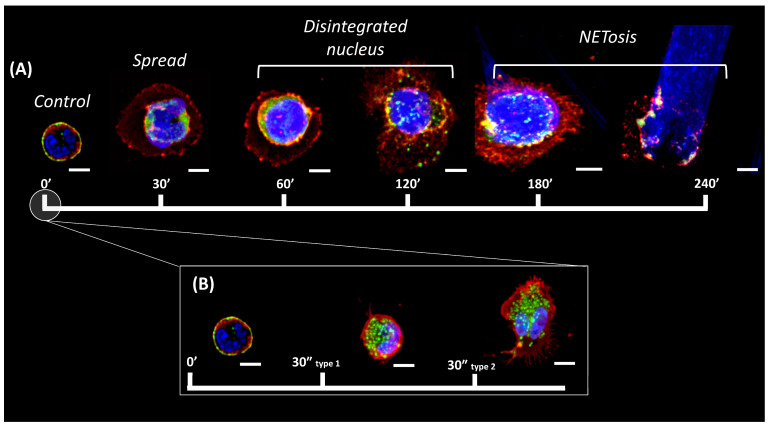
Time periods of neutrophil activation. (**A**) Characteristic groups of neutrophils observed from 0 to 240 min. Control—control neutrophil with a rounded shape, cortical actin and a segmented nucleus. Spread—neutrophil with a membrane spread over the surface, marginal distribution of actin, and a segmented nucleus. Disintegrated nucleus—the nucleus has lost its segmentation and merged into one that fills the cell volume. NETosis—membrane and actin are unchanged, the nucleus extends beyond the edges, and a characteristic ejection appears. (**B**) Characteristic cell types 30 s after cell activation. Red, cell membrane glycoproteins stained with WGA (wheat germ agglutinin) + AlexaFluor 594; green, actin filaments stained with phalloidin + AlexaFluor 488; blue, cell nucleus stained with Hoechst 33342. Images taken with 63×/1.4 oil immersion objective. Scale bar: 5 μm.

To assess the primary response of neutrophils to activator exposure, cells were fixed 30 s after incubation with A23187 and PMA (Figure 1A,B and Figure 2B). This process was not associated with entropic swelling of the nucleus and occurred significantly faster than chromatin decondensation and will be discussed separately in Section 2.

Further cell activation was stopped at time intervals of 30 to 60 min by the addition of a fixative, after which fluorescence imaging of the cells was performed and their size and nuclear changes were assessed over several hours (Figure 1A,B and Figure 2A).

Five groups of neutrophils were examined. Cell membrane glycoproteins were stained with wheat germ agglutinin conjugate to evaluate the cell membrane characteristics, actin filaments were stained with phalloidin conjugate to evaluate the actin cytoskeleton and DNA dye was used for nucleus evaluation. Group 1 (control) neutrophils were characterized by a small size, a rounded shape, and a segmented nucleus (Figure 2A, control). Group 2 consisted of spread polymorphonuclear cells (Figure 2A, spread). Group 3 consisted of spread cells with a disintegrated nucleus (Figure 2A, disintegrated nucleus), group 4 included destroyed cells with NET release (Figure 2A, NETosis), while group 5 cells could not be assigned to any of the groups.

**Figure 3:**
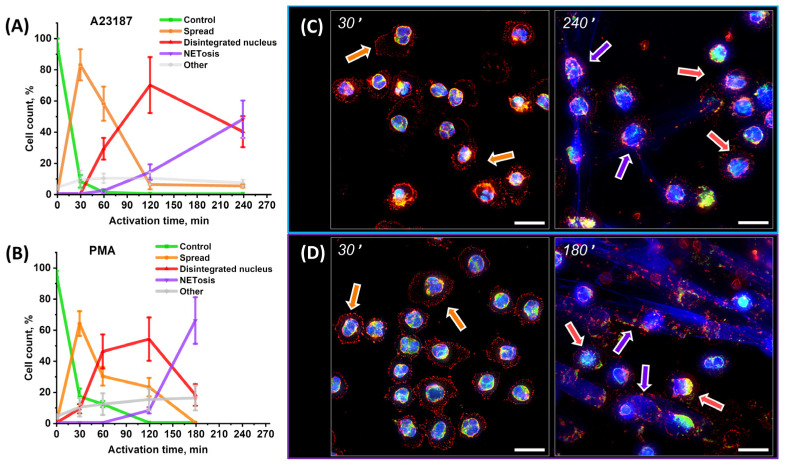
(**A**,**B**) Statistical data on changes in cell morphology during activation. Data are presented as means ± SD. Green line indicates intact and segmented neutrophils. Orange line indicates spread polymorphonuclear cells. Red line indicates neutrophils with disintegrated nucleus. Purple line indicates neutrophils that released NETs. N = 150 cells. (**C**,**D**) Widefield fluorescence images after A23187 (**C**) (time points 30′ and 240′) and PMA (**D**) (time points 30′ and 180′) activation. Orange arrow indicates spread polymorphonuclear cells. Red arrow indicates neutrophils with disintegrated nucleus. Purple arrow indicates neutrophils that released NETs. Red, WGA + AlexaFluor 594; green, phalloidin + AlexaFluor 488; and blue, Hoechst 33342. Images were taken with a 60× oil immersion objective. Orange arrows indicate spread cells, red arrows indicate disintegrated nucleus, and purple arrows indicate cells with NETosis. Scale bar: 20 μm.

After addition of the activator, the number of control cells decreased. Regardless of the activator, control cells were almost completely transformed in 30 min, representing less than 20% of the total number (Figure 3A,B). After 30 min, vesicles also appeared when exposed to A23817 and PMA (Figure 3C,D, orange arrows). The process of neutrophil spreading is described in more detail in Section 2.

After spreading, nuclear swelling began (Figure 3C,D, purple arrows, disintegrated nuclei), which is described in detail in Section 3. NETosis was observed earlier with A23187 activation rather than with PMA activation. For example, at 60 min in A23187, NETosis had already started in some cells (2%), while there were no such cells in PMA-activated cells. At 120 min, A23187 already had 14% NETotic cells versus 8% for PMA (Figure 3A,B). However, NETosis developed more rapidly with PMA activation, with 66% NETotic cells at 180 min versus 48% at 240 min with A23187 activation.

### 2.2. Control Neutrophil Response to Exposure to Activators (The Question Is, How Does a Neutrophil Change from Intact to Activated?)

After establishing time frames for neutrophil changes (as shown in Figure 3), the specific steps were further analyzed and changes identified with confocal images were quantified.

**Figure 4:**
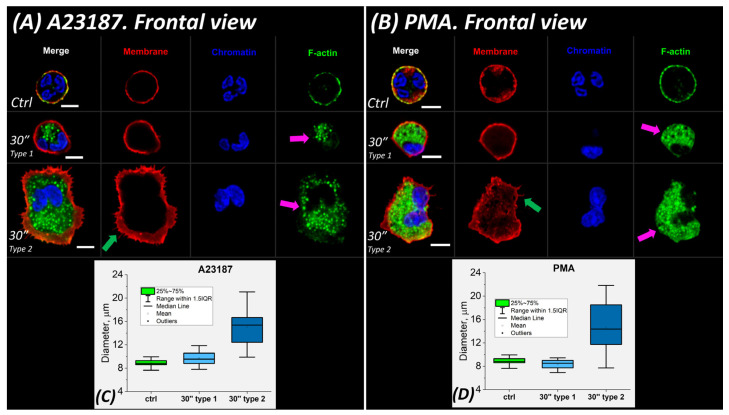

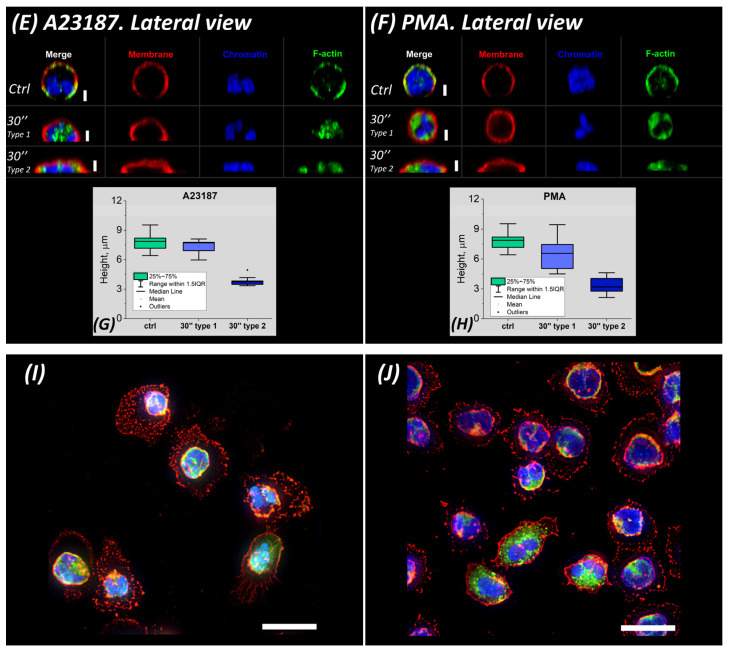
Early stage of neutrophil activation. (**A**,**B**) Images obtained 30″ after activation. Frontal view, A23187 and PMA activation, respectively. Scale bar: 5 μm. Ctrl, intact cells before addition of activator. 30″ type 1 response with actin cytoskeleton rearrangement in neutrophils. 30″ type 2 response with neutrophils spreading on the glass and their size increasing significantly. (**C**,**D**) Box plots of cell diameters. (**E**,**F**) Lateral view, A23187 and PMA activation, respectively. Scale bar: 3 μm. (**G**,**H**) Box plots of cell heights during A23187 and PMA activation, respectively. (**I**,**J**) Images taken 30 min after activation. Frontal view, A23187 and PMA activation, respectively. Scale bar: 20 μm. Red, WGA + AlexaFluor 594; green, phalloidin + AlexaFluor 488; blue, Hoechst 33342. Images taken with a 63×/1.4 oil immersion objective. Pink arrows indicate globular actin structures. Green arrows indicate pseudopodia.

The control neutrophil was a neutrophil isolated by the described procedure prior to the addition of activators. The membrane was spherical, with a characteristic cell diameter of 8.84 ± 0.55 μm and a height of 7.81 ± 0.80 μm. Actin was distributed along the perimeter of the membrane. The nucleus maintained its segmented structure (Figure 4A,B,E,F).

The first step was exposure to activators. To capture the response of neutrophils after exposure to activators, they were fixed 30″ after the addition of A23187 and PMA. There was a reorganization of the actin cytoskeleton and a change in the shape of half of the cells (48% for the activator A23187 and 54% for PMA).

Two types of responses were observed: 30″ type 1 (24% for A23187 and 30% for PMA) and 30″ type 2 (24% for both activators).

The 30″ type 1 was characterized by preserved spherical shape of neutrophils even when activated by A23187 and PMA. The cell diameter and height either remained at the level of control cells or changed insignificantly by no more than ± 15% of control values (Figure 4C,D,G,H).

F-actin changed its configuration compared to controls, moving from the cell periphery to the center of the cytoplasm. This change in f-actin was observed upon both A23187 and PMA activation. The new actin structure consisted of a cluster of small “grains” (Figure 4A,B, pink arrows). The size of individual grains was 0.55 ± 0.12 μm for A23187 and 0.48 ± 0.16 μm for PMA. The grains were distinguishable on both the frontal (Figure 4A,B) and lateral projections (Figure 4E,F).

The 30″ type 2 cells differed from both the control and the 30″ type 1 cells. Neutrophils lost their spherical shape and spread randomly on the surface. Pseudopodia appeared at the edges of the membrane (Figure 4A,B, green arrows). The cell diameter increased significantly compared to 30″ type 1, by approximately 1.55-fold (15.08 ± 3.07 μm) for A23187 and approximately 1.75-fold (14.62 ± 4.36 μm) for PMA. At the same time, the cells approximately halved in size (48%) for both activators compared to 30″ type 1 (Figure 4C,D,G,H). Actin retained its granular structure but was distributed throughout the cell volume, unlike 30″ type 1 cells (Figure 4A,B,E,F).

The main difference between 30″ type 1 cells and 30″ type 2 cells was that the former retained their control shape, while the latter spread out on the glass. However, the volume of the cells remained almost unchanged; an increase in the area of 30″ type 2 cells was compensated by a decrease in their height. It was characteristic for both type 1 and type 2 cells that nuclear segmentation was preserved upon exposure to both activator A23187 and activator PMA. Type 2 cells were still observed after 30 min (Figure 4I,J) together with the spread segmented cells. Therefore, the transition of type 2 cells to spread cells may occur over a long time interval or not at all. The granular structure of actin was observed in both cell types. The rearrangement of f-actin plays a key role in neutrophil spreading, and the likely mechanisms of the actin effect are discussed in detail in the Section 3.

The fundamental difference between the initial response of neutrophils and further activation processes was that the response to activator exposure occurred in a very short time, about 10–60 s. This time is 300–500 times shorter than the studied period of neutrophil activation and NETosis (150–250 min). In fact, the described reaction had an explosive character and led to deformation of neutrophils. Similar effects have been described previously [31,32].

### 2.3. Decondensation of Chromatin and Release of NETS (The Questions Are, What Causes Changes in the Membrane and How Does the Cell and Nuclear Volume Change?)

**Figure 5:**
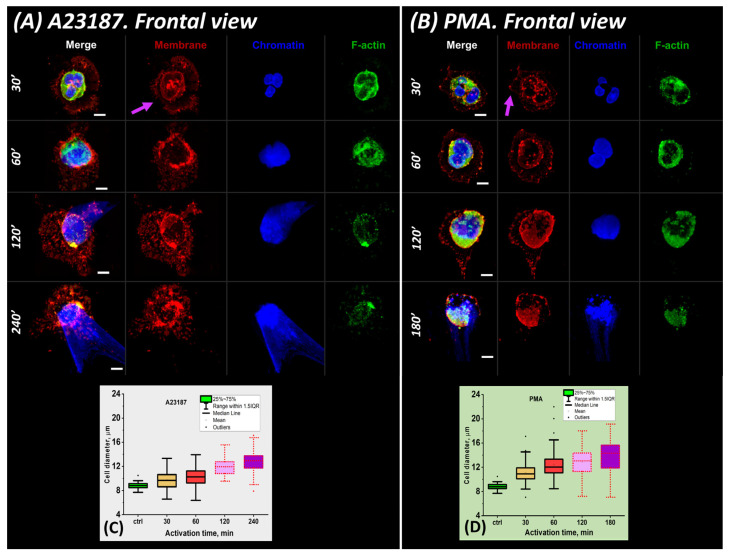

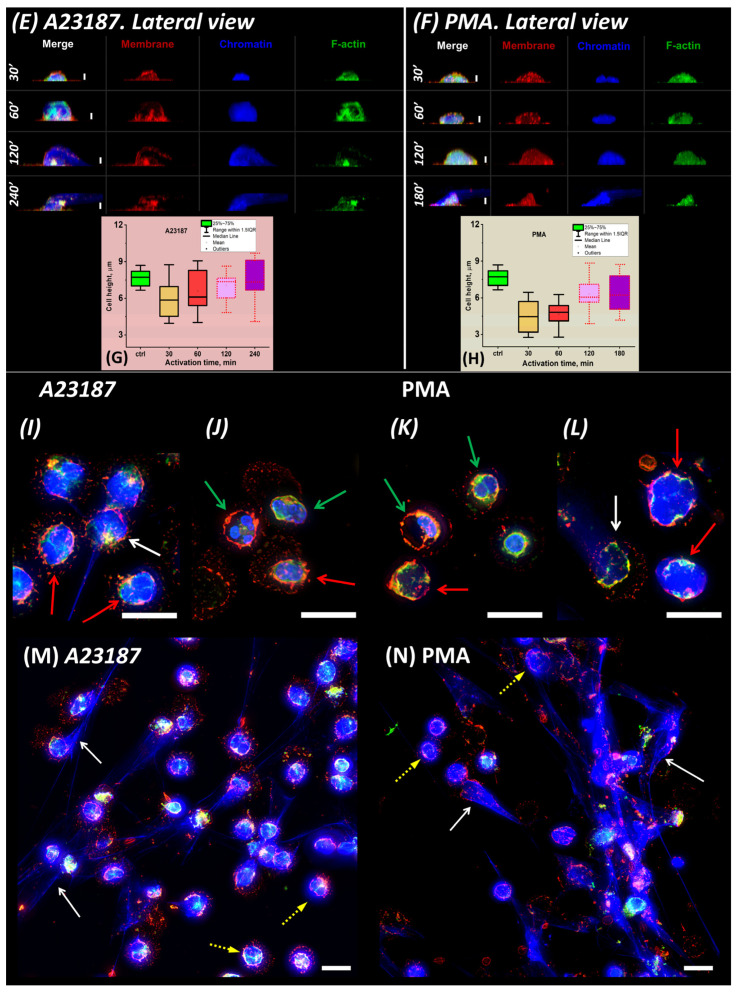
Phases of neutrophil changes associated with nuclear swelling visualized by CLSM. (**A**,**B**) Frontal view of A23187 and PMA activation, respectively. Scale bar: 5 μm. (**C**,**D**) Box plots of cell diameters. (**E**,**F**) Lateral view of A23187 and PMA activation, respectively. Scale bar: 3 μm. (**G**,**H**) Box plots of cell heights. (**I**–**L**) Characteristic cells at late stage of NETosis 120′. A23187 and PMA cell activation, respectively. Scale bar: 20 μm. (**M**,**N**) NETosis in a group of cells (180′ and 240′ after addition of activator). Scale bar: 20 μm. Violet arrows, vesicles; green arrows, spread polymorphonuclear cells; red arrows, disintegrated nuclei; white and yellow dotted arrows, NETosis. Red, WGA + AlexaFluor 594; green, phalloidin + AlexaFluor 488; blue, Hoechst 33342. (**A**,**B**,**E**,**F**) Images obtained with a 63×/1.4 oil lens. (**I**–**N**) Images taken with a 60×/1.4 oil lens.

Further processes of cell activation such as spreading, decondensation of chromatin, swelling of nuclei, rupture of membranes, and release of NET traps were recorded at fixed time intervals. Activation was stopped by addition of fixative at 30, 60, 120, 180, and 240 min. Neutrophils were assessed by fluorescence confocal microscopy, and their size and changes in parameters were evaluated over several hours (Figure 5).

#### 2.3.1. 30 min after Activation

Thirty min after activation, neutrophils were predominantly in the spreading state (Figure 5A,B), with 83% for A23817 and 64% for PMA. The diameter of such spread cells decreased by 25 to 30% (to 10.51 ± 1.15 μm for A23187 and to 10.77 ± 1.18 μm for PMA) compared to 30′ type 2. Correspondingly, their height increased to 5.56 ± 1.04 μm (46%) for A23187 and to 4.72 ± 0.85 μm (40%) for PMA (Figure 5C,D,G,H).

Actin was distributed along the periphery of the membrane. Vesicles formed by membrane structures (violet arrows) were released from the cell, which was a component of the activation process. The vesicles formed a kind of “halo” around the cell that persisted until the end of the observation. At 30′, the nucleus still retained its segmented structure.

Further changes in the morphological parameters of the cell were associated with chromatin decondensation and nuclear swelling.

#### 2.3.2. 60 min after Activation

With the onset of neutrophil entropic swelling, the nucleus lost its segmented shape and individual parts of the nucleus fused into a single structure (Figure 5A,B). The cell diameter increased by 6 to 12% for both activators (Figure 5C,D) and a gradual increase in cell height began (Figure 5E–H). This trend continued until the end of the observation period.

#### 2.3.3. 120 min after Activation

There were more neutrophils whose nucleus continued to swell to the size of the cell membrane, occupying almost all of the available cytoplasmic volume (up to 70% of the total number of cells for A23187 and 54% for PMA) (Figure 5I–L, red arrows). NETosis began to occur in individual neutrophils (Figure 5I–L, white arrows and yellow dashed arrows). In addition, neutrophils with enlarged but segmented nuclei still remained in the solution (up to 6% of the total number of cells for A23187 and 23% for PMA) (Figure 5I–L, green arrows).

Neutrophils increased in size compared to 60′. The increase in diameter reached 11.86 ± 1.31 μm for A23187 and 12.85 ± 2.39 μm for PMA and the increase in height reached 7% for A23187 and 35% for PMA (Figure 5C,D,G,H).

#### 2.3.4. 120–180/240 min after Activation

During this interval after activation, intense chromatin decondensation and NET trap release occurred. Some neutrophils were greatly swollen, while others released extracellular material and shrank. This explains the large variation in neutrophil size in these time intervals. NETosis in most cells for the PMA activator occurred at 180 min, but for A23187, it began only after 240 min (Figure 5M,N). The membrane was severely disrupted and nuclear material was released into the extracellular space. The cell size was virtually unchanged.

To describe the processes occurring in neutrophils after spreading on glass, we introduced two parameters to describe the volume occupied by chromatin (further abbreviated as chr) and the cell volume: V_chr_ and V_cell_. V_chr_ was estimated by Hoechst 33342 and V_cell_ by membrane contour measurement (Alexa Fluor 594-conjugated WGA). These parameters were evaluated using z-stacks observed via a confocal microscope.

In the control, V_chr_ and V_cell_ were 104.77 ± 14.04 μm^3^ and 282.56 ± 48.03 μm^3^, respectively. That is, the chromatin volume was approximately 0.3–0.4 of the cell volume. At 60 min after A23817 activation, V_chr_ increased approximately 2-fold (to 204.74 ± 135.55 μm^3^) and V_cell_ increased 1.3-fold (to 371.28 ± 117.32 μm^3^). The V_chr_/V_cell_ ratio was as high as ~0.6. After PMA activation, the V_chr_/V_cell_ ratio was ~0.5 (191.46 ± 59.40 μm^3^ and 414.73 ± 93.33 μm^3^).

Chromatin began to decondense in the cells, but had not yet left the membrane edges.

V_chr_ and V_cell_ in the late stages of activation (>120′) were difficult to assess due to severe cell damage. The evolution of chromatin swelling was similar for both A23187 and PMA. The increase in volume occupied by chromatin continued throughout the activation period. In the case of A23187, the increase in V_cell_ slowed down or decreased (PMA) as activation progressed, indicating severe membrane degradation.

#### 2.3.5. Patterns of Neutrophil NETosis Imaging

Approximation of the experimental data helps to estimate the time of NET release, i.e., the time when the following is fulfilled:

(1) $$V_{\mathrm{chr}}={\;V}_{\mathrm{cell}}$$

When this condition is met, chromatin completely fills the entire available volume of the cell, after which chromatin leaks out into the extracellular space and NETosis begins.

It was not possible to accurately track the time interval when condition (1) was met for all neutrophils at the same time. There are approximately 2 × 10^5^ cells in the sample at any one time, and activation evolves differently in each cell. Therefore, the timing of NETosis is subject to statistical laws. This is shown in the cell group image (Figure 5), where M is for the activator A23187 at 240 min of activation and N is for PMA at 180 min of activation. Simultaneously, two types of NETotic cells were detected in the sample: (1) cells with a cone-shaped “shoot” (Figure 5M,N, white arrows) and (2) cells in which the NETosis was not a cone-shaped shoot, but the chromatin spread around the cell forming a “cloud” (Figure 5M,N, yellow dotted arrows). Such cells were detected for both A23187 and PMA. The number of such NETosis images is significantly lower than that of cone-shaped shoot cells (~10–20% of the total number of NETotic cells). To describe these simultaneous processes, we selected those with the highest probability of occurrence in a given time interval (the highest number of cells). Under the conditions of our experiments, the most probable interval of NETosis for the PMA activator was 240 min, while for A23187 it was 180 min. After NET release, the increase in chromatin volume V_chr_ was such that:

(2) $$V_{\mathrm{chr}}\;\geq {\;V}_{\mathrm{cell}}$$

**Figure 6:**
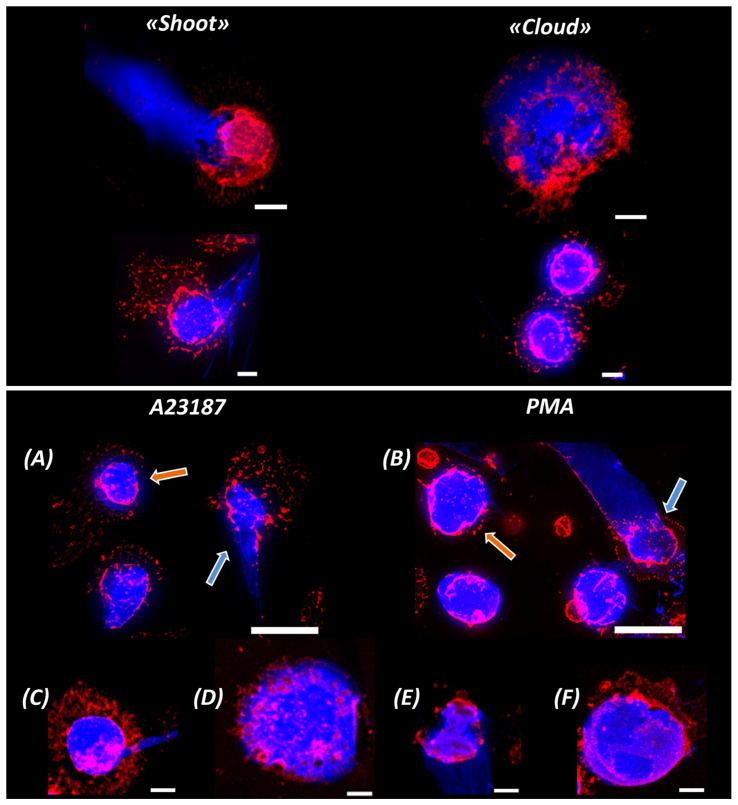
Upper part of the image: characteristic images of NETotic “shoot” and “cloud” cells. Scale bar: 5 μm. (**A**,**B**) 240′ after activation by A23187 and 180′ after activation by PMA. Scale bar: 20 μm. Blue arrows indicate “shoot” chromatin ejection, orange arrows indicate “cloud” chromatin ejection. (**C**,**D**) 240′ after activation by A23187. (**C**) “shoot” cells, (**D**) “cloud” cells. (**E**,**F**) 180′ after activation by PMA. (**E**) “shoot” cells, (**F**) “cloud” cells. Scale bar: 5 μm. Red indicates WGA + AlexaFluor 594, blue indicates Hoechst 33342.

This condition was met in the group image of neutrophils (Figure 5M,N) and in the images of individual neutrophils (Figure 6).

NET trap ejection is a complex explosive process that can follow several pathways. A variety of forms of ejected NET structures have been reported in the literature using electron, confocal, and fluorescence microscopy [1,33,34]. In our study, we distinguish the two most characteristic types of NET traces in the extracellular environment.

The “shoot” mechanism results from the fact that, as a result of an increased chromatin pressure, a certain region of the plasma membrane is destroyed, ruptures, and shoots a DNA network through the resulting pore into the extracellular environment. The size of the pore and the configuration of the ejection track recorded in the image vary greatly from cell to cell and depend on the state of the cell structures at the moment of membrane rupture (high chromatin overpressure, membrane integrity, F-actin structure, and others).

This mechanism produces a characteristic shoot-like trail of released material (Figure 5M,N white arrows, Figure 6A,B blue arrows, and Figure 6A,C,E).

Another type of NETosis presentation is the “cloud” pattern seen in Figure 5M,N (yellow dotted arrows), Figure 6A,B (orange arrows), and Figure 6D,F. In this case, the intracellular material is not “shot out”, but flows around the cell, forming a characteristic cloud (blue) around the cell. The cell membranes are already destroyed at this point, and the network material is released into the external environment at low intracellular pressures. Condition (2) is fulfilled in all shown fragments.

The differences in the images of network ejection are primarily caused by the extent of cell membrane degradation, the rate of chromatin swelling, excessive pressure in the cytoplasm, and other cell parameters.

**Figure 7:**
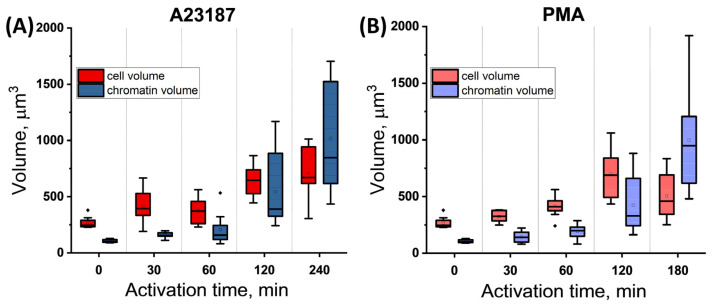
Graph of changes in chr volume and cell volume versus time of neutrophil activation. The data are presented as a box plot. Shades of red are cell volume and shades of blue are chromatin volume.

The processes of activation by A23187 and PMA are illustrated by box plots of chromatin volume and cell volume as a function of activation time according to confocal microscopy data, taking into account the scatter of empirical values (Figure 7).

For A23187, both V_chr_ and V_cell_ grow slowly with little scatter up to 60 min, and the cell volume is always larger than the chromatin volume (Figure 7A). At around 120 min, NETosis begins in some cells and the scatter of V_chr_ values increases, but the average V_chr_ values are still significantly lower than those of V_cell_. At 240 min, the scatter of V_chr_ values increases sharply with the increase in volume, and condition (2) is already met. This occurs as a result of the release of chromatin and cytoplasmic material into the extracellular medium and their outward spread.

Similar trends were observed for the PMA activator (Figure 7B).

The V_cell_ parameter showed different trends depending on the activator; upon exposure to A23187, the cell expanded to its maximum volume, whereupon its deformation stopped (Figure 7A). In PMA-induced NETosis, the cell volume reached its maximum value, after which the membrane collapsed and the cell volume decreased (Figure 7B).

The chromatin volume increased approximately 6–8-fold compared to the control after 240 min for A23187 (V_chr_ = 1018.51 ± 491.93 μm^3^) and 180 min for PMA (V_chr_ = 970.96 ± 333.59 μm^3^).

The cell volume barely increased with A23187 activation at 240′ compared to 120′ (V_cell_ = 706.01 ± 219.99 μm^3^). The trend was different with PMA activation. There was a 28% decrease in volume (down to V_cell_ = 459.50 ± 194.23 μm^3^) compared to 180′ versus 120′, as determined by cell membrane disruption.

## 3. Discussion

In our study, we identified the most characteristic stages in the development of neutrophil activation and subsequent NETosis. These stages are in agreement with similar stages described in the literature [21,23]. However, in contrast to the literature, we recorded the early stage of activation, which is the response of neutrophils to exposure to activator during the first 30 s of the process.

The initial response of neutrophils to the addition of activators was fundamentally different from the process of chromatin decondensation, cell swelling, and NET trap release. Several studies have shown that the first minutes of neutrophil activation are accompanied by morphological changes [29,31].

We recorded two characteristic neutrophil responses to activator exposure. Type 1 includes cells that retain their spherical shape with almost no changes in the parameters and morphology. In these cells, F-actin changed its configuration from cortical to granular and was concentrated in the center of the cytoplasm. Type 2 includes cells that spread along the substrate, whose frontal dimensions increased, height decreased, volume was preserved, and membranes were intact. Actin acquired a granular pattern and was distributed throughout the cell volume.

**Figure 8:**
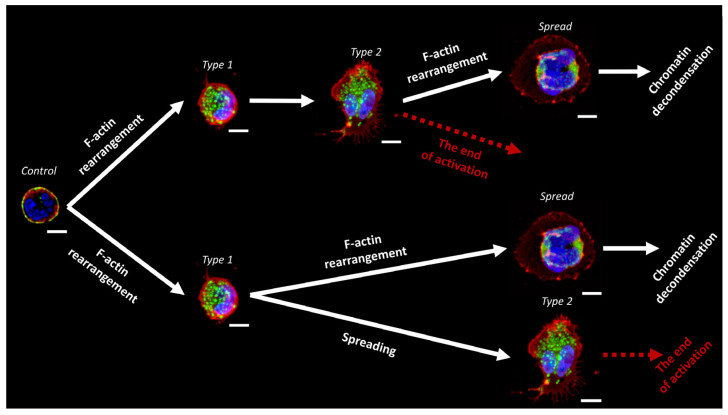
Putative pathways of neutrophil transformation after addition of activator. Scale bar: 5 μm.

It is likely that the type 1 and type 2 cells are “precursors” to the spreading cell (Figure 8). After addition of the activator, actin rearranges into a granular structure that allows the cell to spread on the surface (type 2) (Figure 8, upper branch). Considering that type 2 cells were also observed 30 min after the addition of activators (Figure 4I,J), type 1 and type 2 cells may represent a common stage of activation, after which the cell can either progress towards NETosis or arrest in the type 2 stage (Figure 8, upper branch).

Another possible pathway of NETosis development is shown in the lower branch in Figure 8. After the rearrangement of actin into a granular structure, the cell can evolve into two different types of spreading: “spread” and type 2. Meanwhile, “spread” cells continue to undergo NETosis, while type 2 cells do not change.

There may be several reasons for F-actin rearrangement in type 1 and type 2 cells. Microtubules and vimentin intermediate filaments may be involved in this process [26]. F-actin rearrangement may also be associated with various events that occur during cell adhesion, such as the expression of vinculin protein, which is involved in the regulation of adhesion [35], or the involvement of focal adhesion kinase (FAK) [36].

Similar structures of F-actin have been previously observed in neutrophils [23,29] and other cells at the adhesion stage [37], and a similar membrane transformation has been shown in [23]. Type 2-like cells were also observed when neutrophils were activated by fMLP [31]. That is, after type 2 transformation, the activation of a fraction of the cells slows down, there are no changes in cell morphology, and there is no chromatin ejection.

It is statistically difficult to follow the subsequent behavior of neutrophils of either response type and assess their involvement in further activation and subsequent NETosis. However, such a short and intense process requires further rigorous study.

An ATP-dependent recovery of cortical actin is likely to occur after spreading (the “spread” group). Thirty minutes after the addition of activators, actin no longer rearranges and ultimately degenerates. Further cell deformation is associated with the swelling of the nucleus, which begins its entropic expansion [24] due to the disruption of histone bonds by neutrophil elastase [15,38]. The loss of tight nuclear packing leads to a decrease in nuclear density, filling all the available cell volume and stretching the cell, which ends with membrane destruction and ejection of the networks.

Meanwhile, activated cells have different dynamics of membrane volume changes. The cell membrane can both stretch to its maximum size, after which chromatin extrusion begins, and “burst” into fragments after reaching its maximum size. This can lead to different types of chromatin ejection, i.e., “shoot” and “cloud”.

## 4. Materials and Methods

### 4.1. Reagents

PMA, A23187, Triton X-100, and fetal calf serum (FCS) were purchased from Sigma-Aldrich (St. Louis, MO, USA). Wheat germ agglutinin (WGA)-AlexaFluor 594 conjugate, Phalloidin-AlexaFlour 488 conjugate, and DAPI were obtained from Thermo Fisher Scientific (Waltham, MA, USA). Dextran T-500 was obtained from Pharmacosmos (Holbæk, Denmark). RPMI1640, Hoechst 33342, Ficoll-Hypaque, L-glutamine, and HEPES were obtained from PanEco (Moscow, Russia). Coverslips and 24-well plates were from SPL Co., Ltd. (Pyeongtaek, Republic of Korea). FITC-labeled monoclonal mouse anti-human MPO priming antibodies were purchased from Invitrogen (Waltham, MA, USA).

### 4.2. Isolation of Primary Human Neutrophils

Neutrophils were isolated from heparinized blood as previously described [20]. All experiments were performed in accordance with the guidelines and regulations of the Federal Research and Clinical Center of Intensive Care Medicine and Rehabilitation, V.A. Negovsky Scientific Research Institute of General Reanimatology, Moscow, Russia. All experimental protocols were approved by the institute (protocol no. 2/20 of 10 June 2020). Neutrophil isolation was conducted under aseptic conditions. In brief, blood was gently layered on top of Ficoll-Hypaque (d = 1.077 g/cm^3^) and centrifuged at 400× *g* for 25 min at room temperature (RT). Thereafter, the bulk of the erythrocytes was removed from the suspension by sedimentation with Dextran T-500. The contaminating erythrocytes were lysed in a cold hypotonic sodium chloride solution (0.2%) for 30 s followed by reduction to physiological saline with hypertonic sodium chloride (1.6%). Isolated neutrophils were washed in phosphate-buffered saline (PBS) and suspended in complete medium consisting of RPMI 1640 supplemented with 10 mM HEPES, 2 mM L-glutamine, and 1% heat inactivated fetal calf serum (FCS). Microscopic evaluation of isolated cells revealed that >97% were neutrophils. Cell viability was not <98%, as judged by Trypan blue exclusion. After isolation, neutrophils were resuspended in complete medium at 2 × 10^5^ cells/mL and stored at 4 °C (<1 h) before use in experiments.

### 4.3. Induction and Detection of Neutrophil Extracellular Traps

Freshly isolated neutrophils (2 × 10^5^ cells/mL) were seeded on sterile uncoated round coverslips placed into wells of a 24-well plate in 500 µL complete medium for 30 min at 37 °C. NET formation was induced with 35 nM PMA or 2.5 μM A23187, and coverslips were incubated in the medium for indicated time points at 37 °C and 5% CO_2_. After incubation, coverslips were removed from the first plate and transferred to a second 24-well plate, where the samples were fixed with 4% paraformaldehyde (PFA) for 15 min at RT and washed in PBS 3 times.

### 4.4. Staining Procedure

For immunofluorescence staining, fixed neutrophils were washed with PBS and permeabilized with 0.1% Triton X-100 in PBS for 2 min and RT. Nonspecific binding was reduced by pre-incubation of cells with blocking buffer (including human immunoglobulins) for 20 min. Immunofluorescence staining was performed with FITC-labeled monoclonal mouse anti-human MPO priming antibodies and neutrophil DNA was stained with DAPI (10 µM) for 10 min.

For widefield fluorescence and confocal microscopy, neutrophils were permeabilized with 0.05% Triton X-100 in PBS for 15 min at 4 °C, then washed in PBS and blocked with 3% BSA. For DNA staining, the samples were incubated with Hoechst 33342 dye and diluted in a ratio of 1 to 1000 in PBS for 20 min. For plasma membrane staining, samples were incubated with Alexa Fluor 594-conjugated WGA and diluted in a ratio of 1 to 500 in PBS for 30 min. Alexa Fluor 488-conjugated Phalloidin, diluted in a ratio of 1 to 600 in PBS, was used to stain F-actin. Staining at all stages was carried out in the dark. After staining, coverslips were washed 3 times in PBS and then mounted in Abberior mount solid antifade (Abberior, Göttingen, Germany) on glass slides prior to imaging.

### 4.5. Widefield Fluorescence Microscopy

Widefield fluorescence microscopy was performed on a Thunder microscope (Leica Microsystems, Wetzlar, Germany) equipped with an LED excitation and using 63× oil immersion objective. Tile scans were performed on 8 basic fields for every coverslip. Image processing was performed in LAS X software version 3.0.0.15697 (Leica Microsystems, Wetzlar, Germany).

### 4.6. Confocal Laser-Scanning Microscope (CLSM)

For excitement of Hoechst 33342, a 405 nm laser was used; to excite Alexa Fluor 488 phalloidin, a 488 nm laser was used; and to excite Alexa Fluor 594 WGA, a 543 nm laser was used. Images were acquired with a Zeiss LSM880 confocal laser scanning microscope (Carl Zeiss, Jena, Germany) with Airyscan module using a 63× immersion lens. Image processing, including conversion of imaged z-stacks into maximum intensity projections (MIP), was performed in ImageJ [39]. Z-stacks of 10 single cells were acquired for every time point.

### 4.7. Statistical Analysis

Using a confocal microscope, z-stacks of 10 cells were obtained at each time point. These images were used to estimate the volume of the nucleus, the volume of the cell membrane, and also the cell diameter and height. All measurements were taken using ImageJ software version 1.53q. Volume was evaluated using a 3D object counter. Data on the volume of the nucleus are presented as means ± SD.

Using a widefield microscope, z-stacks of 8 fields were obtained at each time point. These images were used to evaluate cell sizes and cell counts by group. Images were evaluated using LAS X software version 3.0.0.15697. N = 300 cells. The data are presented as means ± SD.

A one-way ANOVA with Bonferroni correction was applied to assess differences between multiple groups. The data are expressed as means ± SEM. All statistical analyses and approximations were performed using Origin Pro 2019 (OriginLab Corporation, Northampton, MA, USA).

## 5. Conclusions

Our findings add to the understanding of conformational changes in NETosis-activated neutrophils, which will allow future studies to develop these processes, especially when documenting cellular events that occur shortly after the addition of activators.

## Data Availability

The datasets used and analyzed during the current study are available from the corresponding authors upon request.

## References

[1] Brinkmann V., Reichard U., Goosmann C., Fauler B., Uhlemann Y., Weiss D.S., Weinrauch Y., Zychlinsky A. (2004). Neutrophil Extracellular Traps Kill Bacteria. Science.

[2] Vorobjeva N.V., Chernyak B.V. (2020). NETosis: Molecular Mechanisms, Role in Physiology and Pathology. Biochemistry.

[3] Papayannopoulos V. (2018). Neutrophil extracellular traps in immunity and disease. Nat. Rev. Immunol..

[4] Pinegin B., Vorobjeva N., Pinegin V. (2015). Neutrophil extracellular traps and their role in the development of chronic inflammation and autoimmunity. Autoimmun. Rev..

[5] Kapoor S., Opneja A., Nayak L. (2018). The role of neutrophils in thrombosis. Thromb. Res..

[6] Porto B.N., Stein R.T. (2016). Neutrophil Extracellular Traps in Pulmonary Diseases: Too Much of a Good Thing?. Front. Immunol..

[7] Chen Z., Zhang H., Qu M., Nan K., Cao H., Cata J.P., Chen W., Miao C. (2021). Review: The Emerging Role of Neutrophil Extracellular Traps in Sepsis and Sepsis-Associated Thrombosis. Front. Cell. Infect. Microbiol..

[8] Grebenchikov O.A., Kasatkina I.S., Kadantseva K.K., Meshkov M.A., Bayeva A.A. (2020). The Effect of Lithium Chloride on Neutrophil Activation on Exposure to Serum of Patients with Septic Shock. Gen. Reanimatol..

[9] Zhao J., Jin J. (2022). Neutrophil extracellular traps: New players in cancer research. Front. Immunol..

[10] Dicker A.J., Crichton M.L., Pumphrey E.G., Cassidy A.J., Suarez-Cuartin G., Sibila O., Furrie E., Fong C.J., Ibrahim W., Brady G. (2018). Neutrophil extracellular traps are associated with disease severity and microbiota diversity in patients with chronic obstructive pulmonary disease. J. Allergy Clin. Immunol..

[11] Pérez-Figueroa E., Álvarez-Carrasco P., Ortega E., Maldonado-Bernal C. (2021). Neutrophils: Many Ways to Die. Front. Immunol..

[12] Schoen J., Euler M., Schauer C., Schett G., Herrmann M., Knopf J., Yaykasli K.O. (2022). Neutrophils’ Extracellular Trap Mechanisms: From Physiology to Pathology. Int. J. Mol. Sci..

[13] Kaplan M.J., Radic M. (2012). Neutrophil Extracellular Traps: Double-Edged Swords of Innate Immunity. J. Immunol..

[14] Fuchs T.A., Abed U., Goosmann C., Hurwitz R., Schulze I., Wahn V., Weinrauch Y., Brinkmann V., Zychlinsky A. (2007). Novel cell death program leads to neutrophil extracellular traps. J. Cell Biol..

[15] Papayannopoulos V., Metzler K.D., Hakkim A., Zychlinsky A. (2010). Neutrophil elastase and myeloperoxidase regulate the formation of neutrophil extracellular traps. J. Cell Biol..

[16] Parker H., Albrett A.M., Kettle A.J., Winterbourn C.C. (2011). Myeloperoxidase associated with neutrophil extracellular traps is active and mediates bacterial killing in the presence of hydrogen peroxide. J. Leukoc. Biol..

[17] Parker H., Winterbourn C.C. (2013). Reactive oxidants and myeloperoxidase and their involvement in neutrophil extracellular traps. Front. Immunol..

[18] Parker H., Dragunow M., Hampton M.B., Kettle A.J., Winterbourn C.C. (2012). Requirements for NADPH oxidase and myeloperoxidase in neutrophil extracellular trap formation differ depending on the stimulus. J. Leukoc. Biol..

[19] Vorobjeva N., Galkin I., Pletjushkina O., Golyshev S., Zinovkin R., Prikhodko A., Pinegin V., Kondratenko I., Pinegin B., Chernyak B. (2020). Mitochondrial permeability transition pore is involved in oxidative burst and NETosis of human neutrophils. Biochim. Biophys. Acta-Mol. Basis Dis..

[20] Vorobjeva N., Dagil Y., Pashenkov M., Pinegin B., Chernyak B. (2023). Protein kinase C isoforms mediate the formation of neutrophil extracellular traps. Int. Immunopharmacol..

[21] Kenny E.F., Herzig A., Krüger R., Muth A., Mondal S., Thompson P.R., Brinkmann V., von Bernuth H., Zychlinsky A. (2017). Diverse stimuli engage different neutrophil extracellular trap pathways. eLife.

[22] Gupta S., Chan D.W., Zaal K.J., Kaplan M.J. (2018). A High-Throughput Real-Time Imaging Technique To Quantify NETosis and Distinguish Mechanisms of Cell Death in Human Neutrophils. J. Immunol..

[23] Neubert E., Meyer D., Rocca F., Günay G., Kwaczala-Tessmann A., Grandke J., Senger-Sander S., Geisler C., Egner A., Schön M.P. (2018). Chromatin swelling drives neutrophil extracellular trap release. Nat. Commun..

[24] Neubert E., Meyer D., Kruss S., Erpenbeck L. (2020). The power from within—Understanding the driving forces of neutrophil extracellular trap formation. J. Cell Sci..

[25] Papayannopoulos V. (2022). Actin powers the neutrophil traps. Blood.

[26] Thiam H.R., Wong S.L., Wagner D.D., Waterman C.M. (2020). Cellular Mechanisms of NETosis. Annu. Rev. Cell Dev. Biol..

[27] Metzler K.D., Goosmann C., Lubojemska A., Zychlinsky A., Papayannopoulos V. (2014). A Myeloperoxidase-Containing Complex Regulates Neutrophil Elastase Release and Actin Dynamics during NETosis. Cell Rep..

[28] Amulic B., Knackstedt S.L., Abu Abed U., Deigendesch N., Harbort C.J., Caffrey B.E., Brinkmann V., Heppner F.L., Hinds P.W., Zychlinsky A. (2017). Cell-Cycle Proteins Control Production of Neutrophil Extracellular Traps. Dev. Cell.

[29] Thiam H.R., Wong S.L., Qiu R., Kittisopikul M., Vahabikashi A., Goldman A.E., Goldman R.D., Wagner D.D., Waterman C.M. (2020). NETosis proceeds by cytoskeleton and endomembrane disassembly and PAD4-mediated chromatin decondensation and nuclear envelope rupture. Proc. Natl. Acad. Sci. USA.

[30] Ding Z., Du F., Rönnow C.-F., Wang Y., Rahman M., Thorlacius H. (2022). Actin-related protein 2/3 complex regulates neutrophil extracellular trap expulsion and lung damage in abdominal sepsis. Am. J. Physiol. Cell. Mol. Physiol..

[31] Watts R.G., Crispens M.A., Howard T.H. (1991). A quantitative study of the role of F-actin in producing neutrophil shape. Cell Motil. Cytoskelet..

[32] Sengupta K., Aranda-Espinoza H., Smith L., Janmey P., Hammer D. (2006). Spreading of Neutrophils: From Activation to Migration. Biophys. J..

[33] Zhao M., Chi H., Sun L. (2017). Neutrophil Extracellular Traps of Cynoglossus semilaevis: Production Characteristics and Antibacterial Effect. Front. Immunol..

[34] Gomez-Lopez N., Romero R., Xu Y., Miller D., Unkel R., Shaman M., Jacques S.M., Panaitescu B., Garcia-Flores V., Hassan S.S. (2017). Neutrophil Extracellular Traps in the Amniotic Cavity of Women with Intra-Amniotic Infection: A New Mechanism of Host Defense. Reprod. Sci..

[35] Wilson Z., Harman M., Hazlett L., Odzer J., Witt H., Franck C., Reichner J., Lefort C. (2018). Vinculin in Neutrophil Adhesion, Motility and Trafficking. FASEB J..

[36] Abaricia J.O., Shah A.H., Olivares-Navarrete R. (2021). Substrate stiffness induces neutrophil extracellular trap (NET) formation through focal adhesion kinase activation. Biomaterials.

[37] Pacini S., Fazzi R., Montali M., Carnicelli V., Lazzarini E., Petrini M. (2013). Specific Integrin Expression Is Associated with Podosome-Like Structures on Mesodermal Progenitor Cells. Stem. Cells Dev..

[38] Tokuhiro T., Ishikawa A., Sato H., Takita S., Yoshikawa A., Anzai R., Sato S., Aoyagi R., Arita M., Shibuya T. (2021). Oxidized Phospholipids and Neutrophil Elastase Coordinately Play Critical Roles in NET Formation. Front. Cell Dev. Biol..

[39] Schneider C.A., Rasband W.S., Eliceiri K.W. (2012). NIH Image to ImageJ: 25 years of image analysis. Nat. Methods.

